# Adolescent Heavy Drinking Does Not Affect Maturation of Basic Executive Functioning: Longitudinal Findings from the TRAILS Study

**DOI:** 10.1371/journal.pone.0139186

**Published:** 2015-10-21

**Authors:** Sarai R. Boelema, Zeena Harakeh, Martine J. E. van Zandvoort, Sijmen A. Reijneveld, Frank C. Verhulst, Johan Ormel, Wilma A. M. Vollebergh

**Affiliations:** 1 Faculty of Social and Behavioural Sciences, Utrecht University, Utrecht, The Netherlands; 2 Experimental Psychology, Helmholtz Institute Utrecht University, Utrecht, The Netherlands; 3 Department of Health Sciences, University Medical Center Groningen, University of Groningen, Groningen, The Netherlands; 4 Department of Child and Adolescent Psychiatry, Erasmus University Medical Centre, Rotterdam, The Netherlands; 5 Interdisciplinary Centre for Psychopathology and Emotion regulation (ICPE), University Medical Centre Groningen, University of Groningen, Groningen, The Netherlands; UC Merced, UNITED STATES

## Abstract

**Background and Aims:**

Excessive alcohol use is assumed to affect maturation of cognitive functioning in adolescence. However, most existing studies that have tested this hypothesis are seriously flawed due to the use of selective groups and/or cross-sectional designs, which limits the ability to draw firm conclusions. This longitudinal study investigated whether patterns of alcohol use predicted differences in maturation of executive functioning in adolescence. Additionally, gender was tested as a possible moderator.

**Methods:**

We used data from the Tracking Adolescents’ Individual Lives Survey (TRAILS), which comprises a cohort of 2,230 Dutch adolescents. Maturation of executive functioning was measured by assessing the standardized improvement on each of four basic executive functions (i.e., inhibition, working memory, and shift- and sustained attention) between ages 11 and 19. Participants were assigned to one of six (heavy) drinking groups (i.e., non-drinkers, light drinkers, infrequent heavy drinkers, increased heavy drinkers, decreased heavy drinkers, and chronic heavy drinkers). We conducted linear regression analyses, and adjusted for relevant confounders.

**Results:**

The six drinking groups did not reveal significant differences in maturation between drinking groups. E.g., maturation executive functioning of chronic heavy drinkers in comparison to non-drinkers; inhibition: *B* = -0.14, 95% CI [-0.41 to 0.14], working memory: *B* = -0.03, 95% CI [-0.26 to 0.21], shift attention: *B* = 0.13, 95% CI [-0.17 to 0.41], sustained attention: *B* = 0.12, 95% CI [-0.60 to 0.36]. Furthermore, gender was not found to be a significant moderator.

**Conclusions:**

Four years of weekly heavy drinking (i.e., chronic heavy drinkers) did not result in measurable impairments in four basic executive functions. Thus, regular heavy drinking in adolescence does not seem to affect these basic behavioural measures of executive functioning.

## Introduction

In adolescence, significant increases in alcohol consumption are usually found, with prevalence rates of last month alcohol use rising from 16.1% at age 12 to 84.8% at age 16 in Dutch adolescents [1]. Alcohol drinking in adolescence has been associated with several negative consequences. Of particular concern are the neurotoxic effects of alcohol, since the significant maturation of both brain structure [2] and function [3,4] are assumed to underlie a specific vulnerability of the developing adolescent brain to the adverse effects of alcohol [5]. However, the findings from empirical studies that tried to assess these effects so far remain inconclusive due to a number of methodological pitfalls.

Most studies that compared alcohol abusing adolescents with non-abusing adolescents on a broad range of neurocognitive functions, such as language and general intelligence [6], attention and intelligence [7], learning, memory, and visuospatial functioning [8], are not convincing in their conclusions because of their cross-sectional designs. As a result, the reverse effect of neurocognitive impairments on heavy alcohol use is neglected [9,10]. Furthermore, studies were conducted among adolescents diagnosed with Alcohol Use Disorder (AUD), which limits the generalizability of the findings because this specific group has behavioural problems associated with controlling their behaviour (according to DSM-IV-criteria [11]) and often psychiatric comorbidity [12]. This amplifies the limitations to assessing causal relations. In contrast, population studies have shown almost no significant differences between excessive drinkers and controls on neurocognitive functioning [13,14]. However, these population studies are again limited by cross-sectional designs and small sample sizes. Also, definitions of excessive or heavy drinking are not consistent across the studies.

To the best of our knowledge, only three small scale studies (*n* = 75 and 40 respectively) have analysed the effects of alcohol use on neurocognitive maturation in adolescence using a longitudinal design with pre- and post-measurements of neurocognitive functioning in a general population [15–17]. The results of these studies do not support the damaging effects of alcohol use in adolescence either. One study found differences between heavy drinkers (average drinks per month: 9.9 for girls and 6.1 for boys) and controls on only one out of seven neurocognitive tasks, and this difference was significant for girls only. However, there was a relation between hangover symptoms and sustained attention in boys [16]. Two other studies showed increased brain activation with fMRI measurements (but not in all hypothesized brain areas) in adolescents who transitioned to heavy drinking (drinks per drinking day: 4.2 and 6.1 respectively), while no differences between drinkers and non-drinkers were found on task performance [15,17]. Thus, empirical research on the effects of heavy alcohol use on neurocognitive maturation does not result in undisputed findings and calls for large scale population studies on this subject.

The aim of the present study was to investigate whether adolescent alcohol users show a distinctive maturation of basic executive functions compared to non-drinkers in a large population-based sample. We focus on Executive Functioning (EF), since this set of cognitive control functions mediates the ability to guide and direct behaviour and is therefore essential for success in school and at work [18]. Furthermore, executive functioning is hypothesised to develop specifically during adolescence [4], as it parallels the maturation of the parietal and prefrontal cortices [19]. As a result of this prolonged maturational trajectory, executive functioning is assumed particularly vulnerable to the effects of alcohol. We used four measures of EF, inhibition, working memory, sustained attention, and shift attention, as our main outcomes in this study. We used computerised tasks to assess basics forms of these functions, which allowed us to use the same tasks in both early and late adolescence, and compare maturation of task performance between adolescents with different drinking habits. We conducted a pre-exposure measure of executive functioning at age 11 and follow-up measurement at emerging adulthood (age 19). Furthermore, since girls are supposedly more vulnerable due to differences in neuromaturation, hormonal fluctuations, and alcohol metabolism [20], gender was considered as a possible moderator.

## Methods

### Study design

The present study used data from the first, second (for descriptive statistics only), third, and fourth wave of the TRacking Adolescents’ Individual Lives Survey (TRAILS). This is a prospective cohort study of Dutch pre-adolescents at age 11. The target sample involved children living in the North of the Netherlands, covering urban and rural areas Demographic information from five municipalities for all inhabitants born between October 1, 1989, and September 30, 1990, in two of the municipalities, and October 1, 1990, and September 30, 1991, in the other three was obtained. These children attended 135 schools that were approached for participation, of which 13 refused to participate (excluding 338 children). Next, parents of eligible children were informed about the study and then invited to participate. After excluding 210 children who were unable to participate because of serious health or language problems, we invited 2,935 eligible children and their parents to enter the study. Seventy-six percent of eligible adolescents and their parents agreed to participate and were enrolled in the study at baseline (T1) (*n* = 2,230, mean age 11.1 years, *SD* = 0.56, 49.2% male) (for more details on the procedure see: [21,22]. At baseline, children of lower socioeconomic background, boys, and children with poor school performance were slightly less likely to participate. Participants and nonparticipants did not differ in emotional and behaviour problems (De Winter et al., 2005). At the third (T3) wave (*n* = 1,816, mean age 16.3 years, *SD* = 0.70, 47.7% male), the response rate was 81.4%. At the fourth (T4) wave (*n* = 1,596, mean age 19.2 years, *SD* = 0.57, 46% male), the response rate was 70%.

### Procedure

On the first assessment, trained undergraduate psychology students administered neuropsychological tests in adolescents’ schools or designated testing centres (for more information, see [23]. Participants who were unable to attend these assessments were tested at home. On the first to third assessments, adolescents completed self-report questionnaires in groups in school, supervised by an assistant. Their parents also completed a written questionnaire. On the fourth assessment, most adolescents were no longer in secondary education. Therefore, trained professional interviewers conducted the neuropsychological test battery individually at participants’ homes or in a nearby community centre (for more information, see [3]. Parents and their children were asked to fill out a computerised questionnaire (or, per request, a paper-and-pencil questionnaire). The Dutch Central Committee on Research Involving Human Subjects approved the study. Parents and adolescents’ written informed consent was obtained. The confidentiality of the study was emphasised.

### Measures

#### Alcohol use

*Descriptive statistic*: At T1, adolescents were asked: “How often have you been drinking alcohol (for example, a bottle of beer or a glass of wine)?” up until that time point, on a 5-point scale ranging from 0 to 7 times or more. We dichotomised these answers into ‘never or once’ and ‘twice or more’. At T2-T4, adolescents were asked to report their average drinking habits between the previous data collection wave and the present. They were asked four questions: “On how many week(end) days do you normally drink alcohol” and “On an average week(end) day on which you drink alcohol, how much alcohol (glasses, cans, bottles) do you drink?” By multiplying and adding the answers, average weekly quantity of alcohol use can be computed. A Dutch standard drink contains 10 grams of alcohol. Furthermore, at T2-T4 adolescents were asked how many times they had been drunk in the last 12 months. We dichotomised these answers into never and once or more. These measures of alcohol use were used as descriptive statistics.

*Drinking groups*: Since the legal age for buying alcohol in the Netherlands was 16 years at time of the data collection, we used data from T3 and T4 to determine group assignment. As to be expected, prevalence rates of frequent heavy drinking (see below) at T2 were indeed very small (<3%). For constructing drinking groups we used measures of both quantity and frequency of alcohol use. The average amount of glasses on a weekend day (since previous measurement wave) was used since weekend quantity of alcohol use has been shown to be a useful and specific measure of alcohol use at this age [24]. We furthermore used the question “On how many occasions in the last month have you had an alcoholic beverage to drink?” Group assignment was done in two steps; in a first step, participants were classified into four groups according to their answers to these questions per measurement wave. In the second step, these groups per measurement wave were combined, resulting in six final drinking groups.

In step one, per measurement wave, we started with identifying participants that were *not drinking*; those who indicated that they did not consume alcohol on a regular weekend day. Next, for the respondents who consumed alcohol, we set a cut off score at 6 glasses on a weekend day for boys and 5 glasses for girls [25]. Participants who indicated that they drank alcohol on a regular weekend day, but less than the cut off, were qualified as *drinking*, *not heavy drinking*. For participants scoring above the cut off (i.e., drinking 5–6 glasses or more on a regular weekend day), we looked at frequency of alcohol consumption. Those scoring above the cut off but drinking on an irregular bases (last month prevalence < 4 times heavy drinking) were considered to be *infrequent heavy drinking*. Finally, respondents falling above the cut off and drinking regularly (last month prevalence ≥ 4 times drinking, i.e., weekly drinking) were classified as *heavy drinking*.

In step two, these categorizations per measurement wave were combined into longitudinal drinking groups. Participants were divided into six groups: non-drinker, light drinker, infrequent heavy drinker, increasing heavy drinker, decreasing heavy drinker, and chronic heavy drinker. For example, a participant *not drinking* at T3 and *heavy drinking* at T4 was longitudinally classified as increasing heavy drinker. For all possible combinations and descriptive statistics, see Table 1.

**Table 1:** Descriptive Statistics of the Drinking Groups.

	Non-drinkers	Light drinkers	Infrequent heavy drinkers	Increased heavy drinkers	Decreased heavy drinkers	Chronic heavy drinkers
Drinking behaviour at ages 16+19 (possible combinations separated by semicolons)	ND+ND	ND+DNHD;	ND+IHD;	ND+HD;	HD+ND;	HD+HD
		DNHD+ND;	DNHD+IHD;	DNHD+HD;	HD+DNHD;	
		DNHD+ DNHD	IHD+ND;	IHD+ND	HD+IHD	
			IHD+DNHD;			
			IHD+IHD			
N	85	873	272	514	250	232
% male	34	48	39	58	47	54
Age at baseline	11.3^a^	11.4^a^	11.4^a^	11.4^a^	11.4^a^	11.4^a^
Parent SES at baseline	-0.09^a^	-0.01^a^	-0.15^a^	0.01^a^	-0.18^a^	-0.08^a^
Maternal alcohol use (SD)	2.5(3.7)^a^	3.3(4.1)^a,b^	3.0(4.0)^a,b^	3.7(4.4)^b,c^	3.7(4.4)^b,c^	4.5(5.1)^c^
Paternal alcohol use (SD)	4.4(5.0)^a^	5.1(5.3)^a,b^	5.1(5.3)^a,b^	6.2(5.6)^b,c^	6.0(5.8)^b,c^	6.7(5.9)^c^
Delinquent behaviour age 11(SD)	0.19(0.18)^a^	0.21(0.15)^a,b^	0.22(0.16)^a,b^ *	0.24(0.17)^a,b,c^	0.26(0.18)^b,c^	0.28(0.22)^c^
Prevalence last year cannabis use age 16 (%)	7^-^	33^-^	42	45	62^+^	70^+^
Prevalence last year cannabis use age 19 (%)	2^-^	40^-^	44	61^+^	58	67^+^
Prevalence daily smoking at age 16 (%)	10^-^	16^-^	29	27	44^+^	48^+^
Prevalence daily smoking at age 19 (%)	8^-^	21	29	40^+^	42^+^	56^+^
Prevalence of haven drunk >1 glass age 11 (%)	5^†^	12^-^	15	19	21	23^+^
Prevalence last year drunkenness age 13 (%)	9^-^	20^-^	26	28	35^+^	36^+^
Prevalence last year drunkenness age 16 (%)	14^-^	55^-^	74	77	94^+^	98^+^
Prevalence last year drunkenness age 19 (%)	22^-^	74^-^	88	98^+^	82	97^+^
N glasses per week age 13 (SD)	1.0(3.1)^a^	1.1(3.2)^a^	1.4(3.1)^a^	1.5(3.2)^b^	3.1(5.6)^b^	3.1(5.0)^b^
N glasses per week age 16 (SD)	0.1(0.1)^a^	3.1(2.5)^a^	5.8(4.5)^b^	5.8(4.8)^b^	12.9(6.5)^c^ **	14.2(7.1)^c^ **
N glasses per week age 19 (SD)	0.4(2.0)^a^	4.8(3.5)^b^ ***	7.2(5.0)^c^	14.0(7.5)^d^ ****	6.6(5.3)^c^ ***	15.8(8.2)^e^ ****
Inhibition M(SD) age 11 in ms	198 (141)	197(157)	190(170)	197(156)	202(140)	199(148)
Working Memory M(SD) age 11 in ms	443 (268)	479(270)	475(277)	473(256)	480(272)	487(268)
Sustained Attention M(SD) age 11 in sec	1.79(1.05)	1.76(0.94)	1.69(0.85)	1.74(0.92)	1.83(0.93)	1.78(0.89)
Shift Attention M(SD) age 11 in ms	586(245)	559(219)	544(201)	544(210)	556(210)	570(219)
Inhibition M(SD) age 19 in ms	162(141)	173(142)	172(201)	174(142)	196(151)	189(148)
Working Memory M(SD) age 19 in ms	243(175)	240(150)	245(146)	235(143)	257(157)	260(155)
Sustained Attention M(SD) age 19 in sec	0.97(0.51)	0.93(0.44)	0.92(0.42)	0.92(0.49)	0.97(0.47)	0.95(0.41)
Shift Attention M(SD) age 19 in ms	359(132)	334(131)	332(120)	339(134)	351(169)	350(143)
Inhibition standardized change score M(SD)	0.11(1.09)	0.03(1.17)	-0.01(1.17)	0.02(1.19)	-0.10(1.17)	-0.07(1.13)
Working Memory standardized change score M(SD)	-0.12(1.23)	0.03(1.03)	-0.01(1.06)	0.04(1.03)	-0.08(1.03)	-0.08(1.02)
Sustained Attention standardized change score M(SD)	-0.05(0.87)	0.02(0.95)	-0.04(0.85)	0.01(1.11)	-0.01(0.92)	-0.01(0.93)
Shift Attention standardized change score M(SD)	0.00(1.26)	0.06(1.14)	0.00(1.04)	-0.05(1.19)	-0.08(1.31)	-0.01(1.32)

Note: ND = not drinking, DNHD = drinking, not heavy drinking, IHD = infrequent heavy drinking, HD = heavy drinking.

+ or—signs mean Pearson-Chi-Square Test is significant.

+ means cell count is higher than expected,—means cell count is lower than expected (based on significant standardized residuals for all imputed datasets).

†: cell count was lower than 5.

Different superscript letters refer to significant differences (p < .05) in mean scores between groups: if two group scores are labelled with the same letter, the scores of these groups do not differ. If two scores are labelled with different letters, these scores differ.

*: in two of the five imputed datasets, there was a significant difference between increasers and other heavy drinking groups.

**: in one of the five imputed datasets, there was a significant difference between decreasers and chronic heavy drinkers.

***: in one of the five imputed datasets, there was no significant difference between light drinkers and decreasers.

****: in two of the imputed datasets, there was no significant difference between increasers and chronic heavy drinkers.

#### Executive functioning

Baseline executive functioning was examined at T1, using three computerised reaction time tasks from the Amsterdam Neuropsychological Tasks (ANT) [26]. The ANT has proven to be a sensitive and valid tool in non-referred samples [23,27,28], as well as in referred samples of various clinical domains [29–32]. We assessed four basic executive functions: inhibition, working memory, shift attention, and sustained attention, since they have sufficient longitudinal stability [3] and the functions are theoretically hypothesized to be associated with alcohol deficits [7]. The ANT has shown to be applicable for longitudinally assessing these basic executive functions in an adolescent population [3]. In Table 2 a description of the tasks used is provided.

**Table 2:** An overview of the four Executive Functions measured with the Amsterdam Neuropsychological Tasks.

Task	Description (answer device: buttons on computer mouse)	Operationalization
**Sustained Attention—dots**	The discrimination of patterns. The participant is shown 600 pictures with 3, 4 or 5 dots (200 trials of each type of stimulus). The target signal is the one with the 4 dots, and the participant has to indicate whether this target signal is shown in the picture by pressing the mouse button with the dominant index finger (‘yes’) or non-dominant index finger (‘no’). The participant hears a sound when s/he makes a mistake. Primary sustained attention index is fluctuation in tempo.	Reflects the ability to maintain a stable performance over a prolonged period. Measured as within-subject SD, reflecting fluctuation in tempo. A higher fluctuation in tempo indicates low scores on sustained attention
**Memory Search—letters**	Recognition of target letters. The task comprises three parts and each part depicts pictures with four letters. In the first part, consisting of 40 trials, participants have to indicate whether the letter ‘k’ is present in the picture by pressing the mouse button with the either dominant index finger (‘yes’) or non-dominant index finger (‘no’). In the second part consisting of 72 trials, participants have to indicate whether both letters ‘k’ and ‘r’ are present in the picture. In the third part consisting of 96 trials, they have to indicate whether all three letters (‘k’, ‘r’ and ‘s’) are shown in the picture. Half of the trials in each part contain a target. This task provides index for memory search capacity (deterioration in speed as a function of memory load).	The ability to maintain and compare increasing informational load in working memory (WM) was evaluated as the difference in RT between Part 3 (high working memory load) and Part 1 (low working memory load). A higher score indicates a poorer working memory capacity.
**Shifting Attentional Set—visual**	A square jumping randomly left/right on a horizontal bar (containing 10 grey squares). The task consists of three parts. In the first part, one of the ten squares is green and jumping randomly left/right on the horizontal bar. If the green square jumps left, the participant has to press the left mouse button and the right mouse button if it jumps right (fixed compatible stimulus-response (SR) mapping condition) (40 trials). In the second part, one of the ten squares is red and jumping randomly left/right on the horizontal bar. If the red square jumps left, the participant has to press the right mouse button and vice versa (fixed incompatible SR-mapping condition requiring inhibition of prepotent responses) (40 trials). The third part is a combination of the first and second part. The square will randomly jump right/left and will turn green/red. When the square is green after the jump, the participant has to press the button in the same direction while if the square becomes red after the jump the participant has to press the opposite button (random SR-mapping condition, requiring mental flexibility–set shifting) (80 trials).	Inhibition reflects the ability to inhibit an inappropriate habitual response tendency. Inhibition was analysed by subtracting RT to (compatible) responses in Part 1 from (incompatible) responses in Part 2). A higher score indicates low (slow) inhibition of prepotent responses.
		Shift Attention reflects the ability to switch between two competing and unpredictable response sets. Inhibition was analysed subtracting RT to (compatible) responses in Part 1 from compatible responses in Part 3. A higher score indicates less Shift Attention.

The use of computerised tasks guarantees standardised assessment while working with reaction times allows detection of subtle improvements in performance. Working memory, inhibition, and shift attention were calculated by subtracting the performance time of a baseline version from a more difficult version of the task. Performance results reflecting more than 50% errors were coded as missing and imputed because they were assumed to reflect either misunderstood instructions or false computer settings, undermining the validity of the testing. In accordance with previous research on neurocognitive maturation [16], we calculated change scores for each of the four executive functions by computing z-scores for the T1 and T4 measures and subsequently subtracting these scores from each other (T1-T4).

#### Measurement of covariates

In accordance with previous studies [16], age at the first assessment, socioeconomic status (SES), paternal and maternal alcohol use, delinquent behaviour at T1, and smoking and cannabis use at age T3 and T4 were included as covariates if they significantly correlated with the outcomes measure (*p* < .05) (see Table 3 for bivariate correlations). *SES* was assessed using income level, educational level, and occupational level of both parents (occupational level was based on the *International Standard Classification for Occupations* [33]). These five variables were standardised and combined into one scale with an internal consistency of .84 (for more information, see [34]. *Paternal and maternal alcohol use* was assessed at T1 by asking the parent who filled out the questionnaire how much alcohol (s)he drinks per week on average and how much his or her partner drinks. *Delinquent behaviour* was measured at T1 Youth Self Report (YSR) [35], consisting of 15 items (α = .71). *Smoking* was also measured at both T3 and T4 by asking: *“*How many times have you smoked cigarettes during the last month?*”*. *Cannabis use* was assessed at T3 and T4 by asking: “How many times have you used weed (marihuana) or hash during the last year?”.

**Table 3:** The Correlation between the Confounders and Maturation of Executive Functioning.

Controlling variable	Δ Inhibition	Δ Working Memory	Δ Shift Attention	ΔSustained Attention
T1 performance	**.59**	**.52**	**.59**	**.49**
Age at T1	-.04	**-.08**	**-.08**	**-.07**
SES	.05	.02	.04	-.01
Maternal alcohol use	.01	.01	.02	-.01
Paternal alcohol use	.05	-.02	.05	-.02
Delinquency scores	.03	-.01	.01	-.01
T3 last year cannabis use	-.01	-.02	-.01	-.05
T4 last year cannabis use	.01	.00	-.02	-.03
T3 last month smoking	.00	.03	**-.09**	-.04
T4 last month smoking	**-.06**	-.04	**-.09**	**-.08**

Note: Variables that correlated significantly (p< .05) are depicted in bold. These variables were controlled for in the regression analyses.

Δ: difference score T1-T4.

### Data Analyses

Multiple data sets in SPSS 20 were imputed, using fully conditional specification (MCMC) with Predictive Mean Matching because there were missing values due to attrition on both predictors and outcome measures [36]. This approach generated five datasets and one pooled dataset [36].

The six drinking groups were validated by comparing them on alcohol related behaviour, such as prevalence of last year drunkenness and drinking in early adolescence. For variables measuring prevalence, Pearson χ^2^ with standardized residuals was used to identify observed counts that were significantly different (*p* < .05) from expected counts. For continuous variables, MANOVA’s with post-hoc tests were used. For these two specific analyses, the five datasets were analysed separately, as pooled analyses are unavailable for these tests.

To examine the influence of drinking groups on maturation of executive functioning, we conducted four bivariate and multivariate linear regression analyses. Standardised change scores of executive functioning were entered as the dependent variable. Dummy variables of drinking groups were constructed and all dummy variables were entered in the model simultaneously as predictors (non-drinkers served as the reference group). First, we conducted four separate linear regression analyses without confounders. In the second model, we conducted four multivariate linear regression analyses adjusting for control variables. Main effects of drinking groups and drinking patters*gender interaction were entered in separate blocks and interpreted accordingly. To reduce Type 1 error, we set α at < .01. For the regression analyses, data from the pooled dataset were used for interpretation.

## Results

### Descriptive statistics

Descriptive statistics are depicted in Table 1. There were no differences between the groups for baseline executive functioning, SES, and age. Pearson χ^2^ yielded significant differences between groups for prevalence rates of other substance use, drinking in early adolescence, and drunkenness. Non-drinkers and light drinkers scored lower than expected on all variables while the chronic heavy drinkers scored higher on all variables.

A MANOVA indicated significant differences between groups on all continuous measures (*p* < .001). Parental alcohol use and delinquency scores in pre-adolescence were largest in the frequent heavy drinking groups (i.e., increasers, decreasers, chronic heavy drinkers). Also, those who later would be classified as frequent heavy drinkers consumed significantly more alcohol at age 13 than the future non-drinkers, light drinkers, and infrequent heavy drinkers. Chronic drinkers, as to be expected, drank most at ages 16 and 19 with an average weekly consumption finally exceeding 14 glasses. Taken together, the results indicate significant differences between the drinking groups on all measures of alcohol related behaviour, validating our identification of the six drinking patterns. The patterns become more differentiated as the adolescents get older.

### The influence of drinking groups on maturation of executive functioning

Raw scores of baseline (T1) and follow-up (T4) executive functioning and standardized change scores are depicted in Table 1. The results of the bivariate linear regression analyses are depicted in Table 4. Drinking group did not significantly predict maturation of executive functioning from age 11 to age 19 for any of the four measures.

**Table 4:** Standardised maturation of Executive Functioning (T1-T4) predicted by drinking groups (T3-T4) without controlling for covariates.

Inhibition	B	99%CI B	β
Light drinkers vs. non-drinkers	-0.08	-0.35 to 0.18	-.04
Infrequent vs. non-drinkers	-0.12	-0.42 to 0.18	-.03
Increasing vs. non-drinkers	-0.09	-0.37 to 0.19	-.03
Decreasing vs. non-drinkers	-0.22	-0.56 to 0.13	-.06
Chronic vs. non-drinkers	-0.18	-0.51 to 0.14	-.05
**Working Memory**			
Light drinkers vs. non-drinkers	0.15	-0.08 to 0.39	.07
Infrequent vs. non-drinkers	0.11	-0.15 to 0.37	.03
Increasing vs. non-drinkers	0.17	-0.08 to 0.42	.07
Decreasing vs. non-drinkers	0.04	-0.23 to 0.31	.01
Chronic vs. non-drinkers	0.04	-0.28 to 0.32	.01
**Shift Attention**			
Light drinkers vs. non-drinkers	0.06	-0.22 to 0.33	.02
Infrequent vs. non-drinkers	0.00	-0.29 to 0.29	.00
Increasing vs. non-drinkers	-0.05	-0.34 to 0.23	-.02
Decreasing vs. non-drinkers	-0.08	-0.39 to 0.23	-.02
Chronic vs. non-drinkers	-0.01	-0.33 to 0.31	-.00
**Sustained Attention**			
Light drinkers vs. non-drinkers	0.07	-0.15 to 0.29	.04
Infrequent vs. non-drinkers	0.01	-0.23 to 0.26	.01
Increasing vs. non-drinkers	0.06	-0.17 to 0.29	.03
Decreasing vs. non-drinkers	0.04	-0.21 to 0.30	.01
Chronic vs. non-drinkers	0.04	-0.23 to 0.30	.01

The results of the multivariate linear regression analyses are depicted in Table 5. In step 1, T1 baseline performance, gender, and other confounders (see Table 3) were added. For all outcome measures, baseline performance significantly predicted maturation, with a higher initial score (i.e., a less optimal performance at baseline) predicting more maturation. This is to be expected since a less optimal performance leaves more room for improvement. Gender differences were significant for inhibition and shift attention, with boys showing more improvement on these tasks. In step 2, we added drinking groups as dummy variables, with non-drinkers as reference group. This did not significantly improve model fit for any of the measures, and none of the dummy variables predicted maturation of executive functioning compared to the non-drinkers. In step 3, we added drinking group*gender interaction variable. Again, this did not improve model fit and thus, gender did not moderate the effects of drinking groups on maturation of executive functioning.

**Table 5:** Standardised maturation of Executive Functioning (T1-T4) predicted by drinking groups (T3-T4) controlling for covariates.

Inhibition		B	SE B	β
Step 1	T1 Inhibition	4.36***	4.09 to 4.64	.57
	Gender	0.17**	0.08 to 0.26	.05
Step 2	Light drinkers vs. non-drinkers	-0.08	-0.29 to 0.14	-.03
	Infrequent vs. non-drinkers	-0.06	-0.30 to 0.18	-.02
	Increasing vs. non-drinkers	-0.07	-0.29 to 0.16	-.03
	Decreasing vs. non-drinkers	-0.19	-0.48 to 0.09	-.05
	Chronic vs. non-drinkers	-0.14	-0.41 to 0.14	-.04
Step 3	Light drinkers vs. non-drinkers *Gender	-0.09	-0.54 to 0.36	-.03
	Infrequent vs. non-drinkers *Gender	-0.13	-0.63 to 0.37	-.02
	Increasing vs. non-drinkers * Gender	-0.09	-0.56 to 0.38	-.03
	Decreasing vs. non-drinkers * Gender	-0.10	-0.43 to 0.62	.02
	Chronic vs. non-drinkers * Gender	-0.05	-0.46 to 0.56	.01
R^2^ = .35 for Step 1, ΔR^2^ = .002 for Step 2 (n.s), ΔR^2^ = .001 for Step 3 (n.s)				
**Working memory**				
Step 1	T1 Working memory	2.02***	1.86 to 2.19	.52
	Gender	-0.05	-0.13 to 0.04	-.02
Step 2	Light drinkers vs. non-drinkers	0.10	-0.11 to 0.30	.04
	Infrequent vs. non-drinkers	0.05	-0.17 to 0.27	.02
	Increasing vs. non-drinkers	0.12	-0.09 to 0.33	.05
	Decreasing vs. non-drinkers	-0.02	-0.25 to 0.21	-.07
	Chronic vs. non-drinkers	-0.03	-0.26 to 0.21	-.01
Step 3	Light drinkers vs. non-drinkers *Gender	-0.42	-0.84 to 0.00	-.16
	Infrequent vs. non-drinkers *Gender	-0.41	-0.86 to 0.05	-.08
	Increasing vs. non-drinkers * Gender	-0.41	-0.84 to 0.02	-.13
	Decreasing vs. non-drinkers * Gender	-0.35	-0.81 to 0.11	-.08
	Chronic vs. non-drinkers * Gender	-0.33	-0.82 to 0.16	-.07
R^2^ = 27 for Step 1, ΔR^2^ = .003 for Step 2 (n.s), ΔR^2^ = .002 for Step 3 (n.s)				
**Shift Attention**				
Step 1	T1 Shift Attention	3.27***	3.06 to 3.48	.59
	Gender	0.26***	0.17 to 0.34	.11
Step 2	Light drinkers vs. non-drinkers	0.15	-0.07 to 0.37	.06
	Infrequent vs. non-drinkers	0.19	-0.04 to 0.43	.05
	Increasing vs. non-drinkers	0.11	-0.12 to 0.32	.04
	Decreasing vs. non-drinkers	0.10	-0.15 to 0.34	.03
	Chronic vs. Non-drinkers	0.13	-0.17 to 0.41	.03
Step 3	Light drinkers vs. non-drinkers *Gender	0.22	-0.23 to 0.67	.07
	Infrequent vs. non-drinkers *Gender	0.09	-0.44 to 0.61	.02
	Increasing vs. non-drinkers * Gender	0.17	-0.30 to 0.65	.04
	Decreasing vs. non-drinkers * Gender	0.51	-0.03 to 1.06	.09
	Chronic vs. non-drinkers * Gender	0.40	-0.13 to 0.94	.06
R^2^ = .37 for Step 1, ΔR^2^ = .002 for Step 2 (n.s), ΔR^2^ = .004 for Step 3 (n.s)				
**Sustained Attention**			
Step 1	T1 Sustained Attention	0.52***	0.48 to 0.56	.49
	Gender	0.03	-0.04 to 0.11	.02
Step 2	Light drinkers vs. non-drinkers	0.13	-0.06 to 0.32	.07
	Infrequent vs. non-drinkers	0.14	-0.08 to 0.35	.05
	Increasing vs. non-drinkers	0.18	-0.02 to 0.39	.08
	Decreasing vs. non-drinkers	0.14	-0.11 to 0.38	.05
	Chronic vs. non-drinkers	0.19	-0.04 to 0.43	.06
Step 3	Light drinkers vs. non-drinkers *Gender	-0.18	-0.58 to 0.22	-.07
	Infrequent vs. non-drinkers *Gender	-0.19	-0.65 to 0.27	-.04
	Increasing vs. non-drinkers * Gender	-0.11	-0.52 to 0.31	-.04
	Decreasing vs. non-drinkers * Gender	-0.17	-0.63 to 0.28	-.04
	Chronic vs. non-drinkers * Gender	-0.12	-0.60 to 0.36	-.03
R^2^ = .25 for Step 1, ΔR^2^ = .001 for Step 2 (n.s), ΔR^2^ = .001 for Step 3 (n.s)				

Confounders: T1 performance of corresponding measure, gender (all analyses). Age at T1, SES, maternal alcohol use, paternal alcohol use, T1 delinquency scores, T3 last year cannabis use, T4 last year cannabis use, T3 last month smoking, T4 last month smoking (if correlating significantly with the outcome measure (See Table 3).

**: significant at p < .01

***: significant at p < .001.

### Additional analyses

All analyses were also conducted with observed cases only (see S1 and S2 Tables). This did not yield different results from the analyses with imputed datasets; i.e., the results were not significant.

Furthermore, we conducted four additional analyses. First, we were interested if the use of a continuous measure of alcohol consumption resulted in different outcomes. Therefore, we conducted linear regression analyses where the average number of glasses per week at T3, at T4, and the sum of these two measures as predictors. This did not result in significant findings (*p*>.01). Second, we tested whether the findings changed when drinking groups were formed according to the definition of heavy drinking by the National Institute on Alcohol Abuse and Alcoholism (i.e., >3/4 glasses daily and >7/14 glasses weekly for women and men respectively). The analysis did not yield significant results (*p*>.01). Third, non-drinkers may consist of a non-normative group of adolescents; therefore, we also examined whether the findings changed when we used light drinkers or infrequent heavy drinkers as reference group instead of non-drinkers. The results were not significant (*p*>.01). Finally, since there are differences in maturation in executive functioning between males and females, we also undertook separate analyses for boys and girls, instead of using gender as a moderator. The results were not significant (*p*>.01). (Data of additional analyses not presented, information available by first author on request).

## Discussion

Our longitudinal study did not show any measurable differences between groups of alcohol users with respect to maturation of executive functioning, contrary to the hypothesis that the developing adolescent brain is particularly vulnerable to neurocognitive aversive effects of alcohol (e.g.,[5]. The present study found expected maturation on all the executive functioning tests that we used (inhibition, working memory, and sustained and shift attention) [3],–hereby measuring executive functioning over a time span of eight years, i.e., with a pre-exposure measure and after four years of (heavy) alcohol consumption. However, we did not find any differences in maturation between any of the drinking groups, not even of the heaviest drinkers (i.e., drinking every weekend and drinking an average of 15 glasses of alcohol each week) for a period of at least four years in adolescence. Gender did not moderate the relation between drinking groups and maturation of executive functioning, which is in contrast to the previous findings that girls are assumed more vulnerable to the toxic effects of alcohol than are boys [13,37].

Our findings are thus in sharp contrast with the common assumption that alcohol leads to measurable changes in adolescents’ executive functioning, although they appear to be in line with the results from the few existing longitudinal studies in adolescents in the general population that did not find neurocognitive deficits at a behavioural level in heavy drinkers for the vast majority of tasks. This implies that statements on how alcohol affects the adolescent brain apparently suffer from an overgeneralisation of research in clinical groups and an over-interpretation of cross-sectional research. On the positive side, the effect of alcohol on the developing brain does not appear to affect the basic executive functioning in an irreversible and devastating way. Thus, despite the obvious acute effects of alcohol as a toxic substance on the adolescent brain (being drunk, being unable to reason while being intoxicated, having hangovers and headaches), it may seem to be flexible enough to cope with these effects of alcohol at least at the level of behavioural development and neuropsychological maturation. That is not to say that the damaging effects of alcohol can be neglected *as such*. Alcohol use might have an effect on brain functions not tested in the current study or on the level of brain activation patterns (e.g., [13,17,38]. Furthermore, in a recent review paper it was concluded that although there is limited evidence for the effects of alcohol use in adolescence on cognitive functioning per se, there is evidence for alcohol being related to stronger automatic affective towards alcohol cues. Especially adolescents with weak (premorbid) executive functions might be at risk for these processes [39]. As important, heavy alcohol use in adolescents is associated with a large number of other well-established risks, such as developing an alcohol use disorder, driving under influence, and engaging in risky behaviour, including violence and fighting while being intoxicated [40]. Furthermore, our results do not rule out the possibility of irreversible effects of alcohol in the long run, either after continuation of heavy drinking of a longer period of time, or the possibility that adolescent heavy drinking might set the stage for deficits in neurocognitive functioning that would manifest at some point later in life.

### Strengths and limitations

Our approach has several strengths and limitations. Strengths are the large population sample and the longitudinal design. To the best of our knowledge, this study was the first to assess heavy drinking in relation to maturation of executive functioning in a longitudinal population cohort of adolescents, covering about eight years, which is longer than in any of the other previous studies. We controlled for several significant confounders that correlated with our dependent variable. We conducted our study in the Netherlands, where legal drinking age is much younger than for example in the United States and alcohol consumption in adolescence is very common, optimising the chances for finding effects of heavy drinking on maturation of executive functioning.

The first limitation concerns the basic tasks used to measure executive functioning. We measured the behavioural consequences of heavy and regular alcohol use with straightforward reaction time tasks, with each task measuring specific sub-components of executive functioning [3]. This does not entail any knowledge on whether the underlying neuro-anatomy is affected and to what extent. For example, equal task performance in heavy drinkers and controls can still be accompanied by differences in neural activation while performing this task [13,38]. It is unclear what these differences represent, but they suggest that alterations in neural processing do not necessarily appear at a behavioural level [15]. In addition, the possibility exists that more complex neuropsychological tasks might have been more sensitive in picking up such alterations. However, an important advantage of starting with the basics was that it allowed using exactly the same tasks at both age 11 and 19, which is a requirement for finding longitudinal change. More complex and strategy-based tasks usually have more stringent age restrictions, and tasks that are both feasible for early adolescents yet still challenging in late adolescence are difficult to find [41]. Furthermore, using straightforward tasks circumvents the problem of ‘task impurity’. Since more complex tasks are assumed to rely on multiple cognitive processes and their integration [16,18,42], it is difficult to identify processes that are responsible for a suboptimal performance [43]. The reaction time tasks we used were not designed to detect deficits, but are able to detect differences between groups on the level of performance. Our findings indicate no performance differences between drinking adolescents and abstaining peers on basic functions, making deficits in these skills unlikely.

The second limitation could be that drinking groups were constructed manually using self-reported measures of alcohol use, although this is common in longitudinal research on adolescent alcohol use [15,16]. Self-report questionnaires have proved to be reliable for assessing alcohol use in adolescence [44]. In addition, our drinking groups showed good and consistent differentiation on validating measures. Although these concern measures obtained from the respondents themselves and not external or independent validators, these findings are reassuring. Chronic drinkers score highest on all alcohol-related behaviours and reveal a heavy drinking pattern at two consecutive waves, which cover at least four years of regular heavy drinking. We are therefore confident that we have adequately identified the most risky drinkers.

The final limitation of longitudinal designs is that attrition may have biased the findings, with most at-risk participants dropping out, resulting in an underestimation of effects and possible loss of power. However, using multiple imputation, missing data on alcohol use and executive functioning were imputed based on a wide variety of associated variables. This technique improves validity of datasets with missing data [45]. Therefore, in our study, attrition bias is unlikely to explain the absence of significant results.

### Implications

We did not find the effects of adolescent alcohol use on maturation of executive functioning in adolescence. Four years of self-reported weekly heavy drinking did not result in deviancies in behavioural performance on a variety of straightforward executive functioning tasks. However, these finding should not be seen as reassuring about adolescent alcohol use as there are numerous other risk related to heavy drinking, such as developing an alcohol use disorder, driving under influence, and engaging in risky behaviour that include violence and fighting while being intoxicated [40]. Consideration of these risks calls for continuous prevention efforts targeting heavy alcohol use in adolescents.

## Supporting Information

S1 Database Database (SPSS file) for data availability. (SAV)

S1 Table Standardised maturation of Executive Functioning (T1-T4) predicted by drinking groups (T3-T4) without controlling for covariates for observed cases only. (DOCX)

S2 Table Standardised maturation of Executive Functioning (T1-T4) predicted by drinking groups (T3-T4) controlling for covariates for observed cases only. (DOCX)
