# Supplementary material for: Adolescent Heavy Drinking Does Not Affect Maturation of Basic Executive Functioning: Longitudinal Findings from the TRAILS Study
Source: PLoS One. 2015 Oct 21;10(10):e0139186. doi: 10.1371/journal.pone.0139186 (PMC4619383; doi:10.1371/journal.pone.0139186)
Supplement: S1 Table — (DOCX) [file pone.0139186.s002.docx]

S1 Table: *Standardised maturation of Executive Functioning (T1-T4) predicted by drinking groups (T3-T4) without controlling for covariates for observed cases only*

| **Inhibition** | **B** | **99%CI B** | **β** |
| --- | --- | --- | --- |
| Light drinkers vs. non-drinkers | -0.01 | -0.37 to 0.33 | -.01 |
| Infrequent vs. non-drinkers | -0.00 | -0.40 to 0.39 | .00 |
| Increasing vs. non-drinkers | -0.06 | -0.43 to 0.31 | -.02 |
| Decreasing vs. non-drinkers | -0.17 | -0.59 to 0.24 | -.04 |
| Chronic vs. non-drinkers | -0.17 | -0.58 to 0.24 | -.05 |
| **Working Memory** |  |  |  |
| Light drinkers vs. non-drinkers | 0.20 | -0.11 to 0.50 | .06 |
| Infrequent vs. non-drinkers | 0.11 | -0.24 to 0.46 | .04 |
| Increasing vs. non-drinkers | 0.21 | -0.12 to 0.53 | .07 |
| Decreasing vs. non-drinkers | -0.00 | -0.37 to 0.37 | .01 |
| Chronic vs. non-drinkers | 0.05 | -0.31 to 0.41 | .01 |
| **Shift Attention** |  |  |  |
| Light drinkers vs. non-drinkers | 0.07 | -0.28 to 0.43 | .03 |
| Infrequent vs. non-drinkers | 0.06 | -0.34 to 0.47 | .02 |
| Increasing vs. non-drinkers | -0.02 | -0.40 to 0.36 | -.01 |
| Decreasing vs. non-drinkers | -0.02 | -0.45 to 0.40 | -.06 |
| Chronic vs. non-drinkers | 0.01 | -0.41 to 0.42 | .00 |
| **Sustained Attention** | |  |  |
| Light drinkers vs. non-drinkers | 0.13 | -0.15 to 0.42 | .07 |
| Infrequent vs. non-drinkers | 0.05 | -0.27 to 0.37 | .01 |
| Increasing vs. non-drinkers | 0.10 | -0.20 to 0.40 | .05 |
| Decreasing vs. non-drinkers | 0.13 | -0.21 to 0.47 | .04 |
| Chronic vs. non-drinkers | 0.08 | -0.25 to 0.41 | .03 |
