# Supplementary material for: Adolescent Heavy Drinking Does Not Affect Maturation of Basic Executive Functioning: Longitudinal Findings from the TRAILS Study
Source: PLoS One. 2015 Oct 21;10(10):e0139186. doi: 10.1371/journal.pone.0139186 (PMC4619383; doi:10.1371/journal.pone.0139186)
Supplement: S2 Table — (DOCX) [file pone.0139186.s003.docx]

S2 Table: *Standardised maturation of Executive Functioning (T1-T4) predicted by drinking groups (T3-T4) controlling for covariates for observed cases only*

| **Inhibition** | | **B** | | **99% CI B** | | **β** |
| --- | --- | --- | --- | --- | --- | --- |
| Step 1 | T1 Inhibition | 4.39^***^ | | 3.98 to 4.81 | | .59 |
|  | Gender | 0.21^***^ | | 0.08 to 0.34 | | .09 |
| Step 2 | Light drinkers vs. non-drinkers | 0.02 | | -0.26 to 0.30 | | .01 |
|  | Infrequent vs. non-drinkers | 0.06 | | -0.26 to 0.37 | | .02 |
|  | Increasing vs. non-drinkers | 0.01 | | -0.29 to 0.30 | | .00 |
|  | Decreasing vs. non-drinkers | -0.13 | | -0.47 to 0.21 | | -.03 |
|  | Chronic vs. non-drinkers | -0.09 | | -0.42 to 0.25 | | -.02 |
| Step 3 | Light drinkers vs. non-drinkers *Gender | -0.03 | | -0.61 to 0.55 | | -.03 |
|  | Infrequent vs. non-drinkers *Gender | -0.03 | | -0.69 to 0.64 | | -.02 |
|  | Increasing vs. non-drinkers * Gender | -0.02 | | -0.63 to 0.59 | | -.04 |
|  | Decreasing vs. non-drinkers * Gender | 0.16 | | -0.53 to 0.85 | | .02 |
|  | Chronic vs. non-drinkers * Gender | 0.11 | | -0.56 to 0.78 | | .01 |
| R^2^= .37 for Step 1, ∆R^2^=.002 for Step 2 (n.s), ∆R^2^=.001 for Step 3 (n.s) | | | | | | |
| **Working memory** | | | | | | |
| Step 1 | T1 Working memory | 2.06^***^ | 1.82 to 2.30 | | | .53 |
|  | Gender | -0.07 | -0.19 to 0.06 | | | -.03 |
| Step 2 | Light drinkers vs. non-drinkers | 0.16 | -0.10 to 0.42 | | | .08 |
|  | Infrequent vs. non-drinkers | 0.10 | -0.20 to 0.40 | | | .03 |
|  | Increasing vs. non-drinkers | 0.17 | -0.10 to 0.45 | | | .07 |
|  | Decreasing vs. non-drinkers | -0.00 | -0.32 to 0.31 | | | -.00 |
|  | Chronic vs. non-drinkers | -0.00 | -0.31 to 0.30 | | | -.00 |
| Step 3 | Light drinkers vs. non-drinkers *Gender | -0.47 | -1.02 to 0.07 | | | -.18 |
|  | Infrequent vs. non-drinkers *Gender | -0.41 | -1.04 to 0.21 | | | -.08 |
|  | Increasing vs. non-drinkers * Gender | -0.44 | -1.01 to 0.13 | | | -.14 |
|  | Decreasing vs. non-drinkers * Gender | -0.44 | -1.09 to 0.21 | | | -.09 |
|  | Chronic vs. non-drinkers * Gender | -0.25 | -0.88 to 0.39 | | | -.05 |
| R^2^= 27 for Step 1, ∆R^2^=.005 for Step 2 (n.s), ∆R^2^=.003 for Step 3 (n.s) | | | | | | |
| **Shift Attention** | | | | | | |
| Step 1 | T1 Shift Attention | 3.37^***^ | | | 3.04 to 3.37 | .63 |
|  | Gender | 0.25^***^ | | | 0.10 to 0.39 | .11 |
| Step 2 | Light drinkers vs. non-drinkers | 0.15 | | | -0.16 to 1.03 | .06 |
|  | Infrequent vs. non-drinkers | 0.25 | | | -0.41 to 0.97 | .07 |
|  | Increasing vs. non-drinkers | 0.12 | | | -0.25 to 1.00 | .04 |
|  | Decreasing vs. non-drinkers | 0.11 | | | -0.01 to 1.42 | .03 |
|  | Chronic vs. Non-drinkers | 0.16 | | | -0.11 to 1.31 | .04 |
| Step 3 | Light drinkers vs. non-drinkers *Gender | 0.43 | | | -0.22 to 0.61 | .15 |
|  | Infrequent vs. non-drinkers *Gender | 0.28 | | | -0.25 to 0.47 | .05 |
|  | Increasing vs. non-drinkers * Gender | 0.38 | | | -0.10 to 0.57 | .11 |
|  | Decreasing vs. non-drinkers * Gender | 0.71 | | | 0.16 to 1.25 | .13 |
|  | Chronic vs. non-drinkers * Gender | 0.60 | | | 0.06 to 1.14 | .12 |
| R^2^= .42 for Step 1, ∆R^2^=.002 for Step 2 (n.s), ∆R^2^=.005 for Step 3 (n.s) | | | | | | |
| **Sustained Attention** | |  | | |  |  |
| Step 1 | T1 Sustained Attention | 0.51^***^ | | | 0.44 to 0.58 | .48 |
|  | Gender | 0.07 | | | -0.05 to 0.19 | -.02 |
| Step 2 | Light drinkers vs. non-drinkers | 0.21 | | | -0.04 to 0.46 | .11 |
|  | Infrequent vs. non-drinkers | 0.20 | | | -0.08 to 0.48 | .07 |
|  | Increasing vs. non-drinkers | 0.24 | | | -0.03 to 0.50 | .11 |
|  | Decreasing vs. non-drinkers | 0.25 | | | -0.05 to 0.56 | .08 |
|  | Chronic vs. Non-drinkers | 0.25 | | | -0.05 to 0.55 | .08 |
| Step 3 | Light drinkers vs. non-drinkers *Gender | -0.14 | | | -0.66 to 0.38 | -.06 |
|  | Infrequent vs. non-drinkers *Gender | -0.11 | | | -0.70 to 0.44 | -.02 |
|  | Increasing vs. non-drinkers * Gender | -0.10 | | | -0.54 to 0.44 | -.04 |
|  | Decreasing vs. non-drinkers * Gender | -0.15 | | | -0.77 to 0.47 | -.03 |
|  | Chronic vs. non-drinkers * Gender | -0.13 | | | -0.73 to 0.47 | -.03 |
| R^2^= .23 for Step 1, ∆R^2^=.007 for Step 2 (n.s), ∆R^2^=.000 for Step 3 (n.s) | | | | | | |
| Confounders: T1 performance of corresponding measure, gender (all analyses). Age at T1, SES, maternal alcohol use, paternal alcohol use, T1 delinquency scores, T3 last year cannabis use, T4 last year cannabis use, T3 last month smoking, T4 last month smoking (if correlating significantly with the outcome measure (See Table 3).  **: significant at p<.01; ***: significant at p<.001 | | | | | | |
